# Impact of COVID-19 on employment: sociodemographic, medical, psychiatric and neuropsychological correlates

**DOI:** 10.3389/fresc.2023.1150734

**Published:** 2023-07-11

**Authors:** Madison Thompson, Stephen J. Ferrando, Rhea Dornbush, Sean Lynch, Sivan Shahar, Lidia Klepacz, Abbas Smiley

**Affiliations:** ^1^ School of Medicine, New York Medical College, Valhalla, NY, United States; ^2^ Department of Psychiatry, Westchester Medical Center Health Network, Valhalla, NY, United States; ^3^ Department of Psychiatry, Icahn School of Medicine at Mount Sinai, Mount Sinai Beth Israel, New York, NY, United States; ^4^ Department of Psychiatry, Icahn School of Medicine at Mount Sinai, Mount Sinai Hospital, New York, NY, United States; ^5^ Department of Surgery, Westchester Medical Center Health Network, Valhalla, NY, United States

**Keywords:** post-acute sequelae of COVID-19 (PASC), long covid, employment, employment impact, neuropsychiatric outcomes

## Abstract

**Introduction:**

Given the nature of the persistent physical and neuropsychiatric symptoms reported in the literature, among individuals after acute COVID illness; there is growing concern about the functional implications of the Post-Acute Sequelae of COVID-19 (PASC). We aim to evaluate associations of sociodemographic, medical, psychiatric and neuropsychological factors with employment status post COVID-19.

**Methods:**

59 participants were administered a neuropsychiatric assessment and queried about employment status and occupational difficulties months after quarantine. Two levels of comparison were conducted: (1) Those who took time off work (TTO) to those with no time off (NTO); (2) Those who reported occupational performance suffered (PS) to those who did not (PDNS).

**Results:**

TTO vs. NTO exhibited extensive differences across medical, psychiatric and neurocognitive domains. PS vs. PDNS differed on subjective measures of physical and cognitive symptoms, but not on objective testing.

**Conclusion:**

Individuals who took time off beyond COVID-19 quarantine experience persistent physical, psychiatric, subjective and objective neurocognitive burden. In contrast, occupational impairment appears to reflect subjective complaints, but not objective measures. Clinical implications are discussed.

## 1. Introduction

As the SARS-CoV-2 pandemic continues, there have been emerging concerns about the long-term impact of COVID-19, particularly the implications of long-term or persistent symptoms now known as the Post-Acute Sequelae of COVID-19 (PASC), commonly known as “Long COVID”, or “Post-COVID Syndrome.” Patients suffering from PASC describe their symptoms as both chronic and debilitating. As such, there have been calls to action in the popular press and among those in the field for more research investigating the prevalence and characteristics of this sequela (1–3). The World Health Organization has defined PASC “as the illness that persists after confirmed or suspected COVID-19 infection, usually within 3 months of infection onset, and with symptoms or effects that last at least 2 months with no other probable cause.” Extensive literature has characterized PASC’s most commonly reported symptoms; muscle pain, weakness, dyspnea, fatigue, depression, PTSD, anxiety, sleep disturbance and impaired concentration, attention, and memory, among others (4–6). In their meta-analysis, Zeng et al. 2022 estimated that of 1,285,407 participants across 151 studies, one fifth of recovered COVID patients demonstrated psychiatric symptoms 12 months after recovery.

Given the nature of both the physical and neuropsychiatric symptoms reported in the literature, there is growing concern about the functional implications of PASC. One such concern is the impact of PASC on employment. The studies investigating the impact of PASC on patients’ ability to work have fallen into three broad categories: (1) the impact of PASC on employment in patients hospitalized for their acute COVID illness, (2) the impact of PASC on employment in patients not hospitalized for their acute COVID illness, and (3) the impact of PASC on employment independent of acute COVID severity.

A large proportion of the literature focused on patients who had been hospitalized throughout the United States and Europe. Although one might hypothesize that due to increased disease severity, patients might have increased occupational impact, in most studies, a majority of previously employed hospitalized patients had returned to work by time of follow-up. Of hospitalized patients previously employed, 40%–69.1% reported returning to work within 1–7 months of follow-up (5, 7–9). However, other studies reported rates as high as 40% of hospitalized (8) and 44.8% of patients admitted to critical care (7) had not yet returned to work at the time of follow-up. In one new study, among those hospitalized who had not returned to work following discharge, 37.5% of those were currently on sick leave, 15% had been furloughed, and 2.5% were newly retired (9).

Fewer studies have focused on the occupational impact of PASC in those with mild, acute COVID symptoms. One Swedish study compared the difference in symptoms and functional impairment after mild COVID infection between healthcare workers who were continually seropositive to those who were continually seronegative for the SARS-CoV-2 IgG antibody throughout 8 months of follow-up (6). Notably, 11% of the seropositive cohort reported at least 1 severe symptom lasting 8 months or more, in addition to moderate to marked disruption in functional impairment on the patient-reported Sheehan Disability Scale, which compared to only 2% of the seronegative cohort (6). Moreover, 8% of the seropositive cohort reported moderate to marked disruption in their work-life due to long-term symptoms, in contrast to 4% of the seronegative cohort (6).

The literature investigating the employment impact of PASC independent of acute COVID severity, in general, reported that 11.5%–31% of participants were not working at 1–7 months follow-up (4, 10, 11). Of patients who had taken sick leave or paid time off of employment, one study reported that 66.1% of participants were on sick leave 1 month (12), 13.3% were on sick leave for at least 12 weeks (12) and 9% remained on sick leave throughout 4 months of follow-up (12). Others found 70% of participants on paid time off employment for 13 weeks or more (13).

While it is critical to understand the impact PASC has had on people’s ability to return to work, it is equally important to understand how PASC has impacted occupational performance once individuals have returned. Among participants hospitalized for their acute COVID illness, 25% and 5% reported reduced hours or modified job responsibilities upon returning to work following hospital discharge (8, 9). In comparison, 45% of participants who had returned to work in studies independent of acute COVID severity reported requiring reduced hours after their acute illness (4). Likewise, 38.9% of patients across both hospitalized and non-hospitalized groups reported marked impairment upon return to work (10), whereas 8% of patients non-hospitalized with mild symptoms reported the same (6). Consistent with impaired performance at work, studies across varying severity of acute COVID illness reported that 11.8%–50% of patients experienced new or worsening, diminished activities of daily life (8, 10). While there is variability in the data presented, it is clear from the literature above that PASC substantially impacts both people’s ability to return to employment and their occupational performance once they have returned.

As part of an ongoing study of the neuropsychiatric sequelae of PASC, we conducted an in-depth assessment of neuropsychological, medical, and psychiatric status. In addition, we inquired about employment status, time away from work after acute COVID infection, and self-attributed reasons for subjective impairment in occupational performance. From this data, we aimed to address some of the existing gaps in the literature about specific determinants of time away from work and functional impairment while at work. Our primary questions were:

1. What are the patterns and reasons for taking time off of work, as well as the factors that affect current work performance in the months after recovery from acute COVID-19?
2. How do individuals who took time off work beyond COVID quarantine compare to those who took no time off in terms of sociodemographic, medical, psychiatric and neuropsychological factors?
3. How do individuals who are currently working at the time of assessment and who say that their work performance has suffered differ from those who do not feel that their performance has suffered?

## 2. Methods

This study was conducted at New York Medical College/Westchester Medical Center Health Network (WMC Health), in Valhalla, NY. It was approved by the Institutional Review Board of New York Medical College (Protocol #14400) as well as the WMC Health Clinical Research Institute. Data were obtained from the baseline assessment of participants recruited for a longitudinal study of neurocognitive, medical, and psychiatric sequelae of COVID-19. Participants were recruited via social media, flyers, email chains, and word-of-mouth. A subset of patients seeking care for “brain fog” were referred from the WMC Health Post-COVID-19 Recovery Program. All interested persons were screened via telephone to determine eligibility for participation by investigators (SL, SS) based on the following criteria: (1) Age at least 20 years old; (2) documented positive COVID-19 nasopharyngeal test or positive antibody test prior to vaccination; (3) recovered from acute COVID-19 infection as per CDC recommendations (10–20 days after symptom onset and 24 h without fever); (4) completed minimum 8th grade education; (5) fluent in English; and (6) capable of signing informed consent. Persons with a prior diagnosis of a major neurocognitive disorder, traumatic brain injury with loss of consciousness, uncorrected visual/hearing deficits, intellectual disability, or unstable psychiatric symptoms were excluded.

Eligible participants met with the study assessors (SL, SS) who were trained to perform and score the assessment battery by co-PI (RD), a board-certified Neuropsychologist, and were supervised by the study PI (SF). During this visit, signed informed consent was obtained. Participants were compensated $40.00 for their time.

### 2.1. Study Measurements and Instruments

Sociodemographic measures included age, gender, race, relationship status, years of education and current employment.

Employment information collected included employment status pre-COVID illness, time taken off work, length of time away from work, self-reported reasons for taking time off, current employment status, current hours working, FMLA, disability, interest in returning to work, performance at work, self-attributed reasons for impaired performance at work and termination of employment questions. The questions related to employment were adapted from studies investigating determinants of employment in HIV infection (14, 15). Occupational Skill level was classified according to the International Standard of Occupations-08 (ISCO-08) (16).

Medical measures included self-reported medical history, including acute COVID-19 symptoms, treatment, and hospitalization, time since diagnosis and number of non-COVID medical comorbidities. COVID-19 symptom severity at the time of acute infection as well as at the time of the study appointment was determined by score on an instrument adapted from published CDC COVID-19 symptoms, assessing severity (absent, mild, moderate, severe) on 11 COVID-19 symptoms, which is scored from 0 to 33 (17). Participants were also administered the Lawton-Brody Instrumental Activities of Daily Living Scale (IADL) which measures increasing difficulty with practical aspects of everyday functioning on a scale of 0–8 (18), and the 11-item Chalder Fatigue Scale (CFS-11), which measures the severity of both mental and physical fatigue and is scored from 0 to 33. A cutoff score of >21 is considered clinically significant fatigue (19).

Psychiatric measures included pre-COVID-19 psychiatric and substance use disorder (SUD) history, current psychiatric medication use and self-report questionnaires to assess current psychiatric symptoms and disorders. Self-report questionnaires included the Patient Health Questionnaire-9 (PHQ-9), which queries DSM-IV major depression criteria and has a maximum score of 27 (20); the Endicott Quality of Life Enjoyment and Satisfaction Scale (Q-LES-Q), which queries overall life satisfaction in 14 areas and has a raw score range of 0–70 (21); the Post Traumatic Stress Disorder Checklist for DSM-5 (PCL-5) which has a maximum score of 80 (22); and the Generalized Anxiety Disorder-7 (GAD-7) questionnaire, which is scored from 0 to 21 (23). Scores on the questionnaires were categorized based on cut-off values in the medical literature. For PHQ-9, a score of ≥10 may indicate clinically significant depressive symptoms (20); for GAD-7, a score ≥10 indicates clinically significant anxiety symptoms (23); for PCL-5, a score of ≥33 indicates clinically significant PTSD symptoms (22).

The neuropsychological battery consisted of measures assessing specific cognitive domains that have been implicated in other infectious and clinical disease states (24–28). The battery included the Test of Premorbid Function (TOPF), to obtain an estimate of pre-COVID-19 intellectual function (29). Participants also completed the Patient Assessment of Own Function (PAOF), which queries subjective cognitive complaints yielding an average score of 0–5 for memory, language and communication, handedness, sensory-perception, and cognitive/intellectual functioning (30). For the study, the PAOF subscales most associated with everyday cognitive functioning, including memory, language and cognitive/intellectual/executive functioning served as measures of subjective cognitive complaints. Participants were administered neuropsychological tests assessing attention; auditory/verbal and visual immediate and delayed memory; visuospatial and constructional abilities; psychomotor speed; language; and executive function. The battery included the Repeatable Battery for the Assessment of Neuropsychological Status (RBANS) Form A (total and 5 subscale scores) (31) and Montreal Cognitive Assessment (MoCA) (32). RBANS scores were converted to standardized t-scores for analysis and MoCA total and subscale scores were utilized.

Analyses were conducted on a sample of 59 participants across two domains: (1) correlates and predictors of taking time off after the quarantine period, and (2) self-reported difficulties of current occupational functioning among participants currently working at time of assessment. The first domain was subcategorized into two cohorts: those who “Took Time Off” (TTO) and those who did not take time off or the “No Time Off” (NTO). The TTO cohort included individuals who took time off work for their long COVID symptoms beyond the required quarantine period, whereas the NTO cohort, included individuals who returned to work directly after the required COVID quarantine period. We compared sociodemographic characteristics, employment measures, and self-reported reasons for taking time off, medical metrics, psychiatric metrics, and neuropsychological testing metrics.

The second domain, in which we assessed the self-reported difficulties of current occupational functioning in long COVID patients currently working, was similarly was categorized into two cohorts—“Performance Suffered” (PS) and “Performance Did Not Suffer” (PDNS). The PS cohort included individuals who were currently working and previously employed prior to acute COVID infection but felt their occupational performance had suffered upon return from their acute illness due to their long COVID symptoms. The PDNS cohort, in turn, included individuals that were currently working but did not feel their occupational performance had suffered upon returning to work after their acute illness. We again compared the two cohorts on sociodemographic characteristics, employment measures, medical metrics, psychiatric metrics, neuropsychological testing metrics, and self-reported reasons of impaired occupational performance.

Data were analyzed using SPSS software (33). These included descriptive statistics (frequency, mean, standard deviation); Chi-square for group comparisons on categorical variables; independent and one-sample *t*-tests and analysis of covariance (ANCOVA) for group comparisons on continuous variables. Significant group differences in variables such as age and number of medical comorbidities were employed as covariates in group comparisons. Logistic regression with backward elimination was used to identify independent predictors of taking time off work. Variables within each measurement domain that bore the strongest difference between TTO and NTO groups were utilized as independent variables.

## 3. Results

### 3.1. Sociodemographic characteristics of entire sample

This study had a total of *N* = 59 participants recruited from both Post-COVID Recovery Program and community populations with 59.3% recruited in clinic and 40.7% recruited from the community. Participants had an average age of 42 years with 69.5% identifying as female and 30.5% as male. A majority of participants identified as White (71.2%), followed by Hispanic (13.6%), African American (6.8%), Asian/South Asian (6.8%) or, other (1.7%). Most participants (71.2%) indicated they were in a relationship or married, and all participants were living in a home or apartment. Participants had similar levels of education and had comparable occupational skill level as classified by the ISCO-08, with 61% of participants falling under the broad skill level 3 and 4 which is predominantly categorized as professionals.

### 3.2. Employment characteristics

Of participants previously employed prior to COVID illness (Table 1 and Supplementary Figure S1), just over three-fourths reported being currently employed at the time of assessment. Over half of those previously employed reported currently working full time, and 16.9% of participants reported working part-time or less. Of those currently working almost half felt their employment performance had suffered since their return and 18.6% participants reported decreased hours. Ten previously employed participants reported not working at the time of assessment, and nine in ten reported loss of employment. Nine in ten participants previously employed and not working reported wanting to work, and six in ten reported taking steps to return to work.

**Table 1:** Employment data of those who were employed prior to COVID illness.

Variable	Previously employed
	*N* = 59
Employment status	
Took time off, *n* (%)	28 (47.5%)
Currently working, *n* (%)	
Full time	35 (59.3%)
20–40 h	4 (6.8%)
10–20 h	4 (6.8%)
<10 h	2 (3.4%)
Not working, *n* (%)	10 (16.9%)
Lost employment	9 (15.3%)
Want to work	9 (15.3%)
Steps to return to work	6 (10.2%)
Employment performance suffered, *n* (%)	28 (47.5%)
Decreased hours, *n* (%)	11 (18.6%)

Just less than half, of participants took time off work beyond required quarantine period for their persistent COVID symptoms (Table 1 and Supplementary Figure S1). Of those previously employed who took time off (Table 2 and Supplementary Figure S1), most had taken more than 8 weeks off, almost one-third had taken off 4–8 weeks, a quarter had taken off 4 weeks or less and only 7.1% had taken off less than 1 week of work. Just under half of participants who took time off reported using FMLA, while 17.9% reported being on disability. Three-fifths of participants who took time off reported currently working at the time of assessment, almost half of which reported working full time and 14.3% working part-time or less. Fifty percent of participants who had returned to work after taking time off, reported that they felt their employment performance suffered when they returned. About one-third of participants who had taken time off reported not working at the time of assessment, all of which reported wanting to work, and two-thirds reported taking steps to return to work. A quarter of participants who had taken time off work reported decreased hours upon returning to work, while just over one-fifth reported loss of employment.

**Table 2:** Time off work & employment data of those who took time off.

Variable	Took time off
	*N* = 28
Time off	
Time off, *n* (%)	
<1 week	2 (7.1%)
1–4 weeks	5 (17.9%)
4–8 weeks	9 (32.1%)
>8 weeks	12 (42.9%)
Currently working, *n* (%)	
Full time	13 (46.4%)
20–40 h	2 (7.1%)
10–20 h	1 (3.6%)
<10 h	1 (3.6%)
Not working, *n* (%)	9 (32.1%)
Lost employment	6 (21.4%)
Want to work	9 (32.1%)
Steps to return to work	6 (21.4%)
FMLA, *n* (%)	13 (46.4%)
Disability, *n* (%)	5 (17.9%)
Employment performance suffered, *n* (%)	14 (50.0%)
Decreased hours, *n* (%)	7 (25.0%)

The most prevalent self-attributed reasons for taking time away from work were fatigue, concentration, memory and physical symptoms (Table 3 and Supplementary Figures S2, S3). The most highly self-reported reasons for impaired occupational performance were difficulties with fatigue, and motivation, followed by difficulties in attention, memory, concentration, multitasking and slowed thoughts.

**Table 3:** Self-attributed reasons for taking time off work and for occupational performance impairment among those currently working.

Variable	Took time off
	*N* = 28
Self-attributed reasons for taking time off	
Fatigue, *n* (%)	22 (78.6%)
Concentration, *n* (%)	20 (71.4%)
Memory, *n* (%)	19 (67.9%)
Systemic issues, *n* (%)	14 (50.0%)
Other, *n* (%)	9 (32.1%)
Self-attributed reasons for impaired occupational performance after acute illness	Occupational Impairment *N* = 28
Difficulties fatigue, *n* (%)	14 (50.0%)
Difficulties motivation, *n* (%)	12 (42.9%)
Difficulties attention, *n* (%)	11 (39.3%)
Difficulties memory, *n* (%)	11 (39.3%)
Difficulties concentration, *n* (%)	10 (35.7%)
Difficulties multitasking, *n* (%)	10 (35.7%)
Difficulties thoughts slowing, *n* (%)	10 (35.7%)
Difficulties other, *n* (%)	1 (3.6%)

### 3.3. Comparison of time off vs. no time off cohorts

#### 3.3.1. Sociodemographic characteristics

The Took Time Off (TTO) cohort was significantly older than the No Time Off (NTO) cohort, but otherwise did not significantly differ in other sociodemographic characteristics (Table 4). While there were no significant differences between the two groups, the majority of participants in both groups identified as white, female and had similar levels of educational attainment.

**Table 4:** Characteristics of participants who reported taking time off of work beyond required quarantine due to persistent COVID symptoms compared to those who took no time off of work beyond required quarantine.

Variable	Time off	No time off	Statistic	*p* *
	*N* = 28	*N* = 31		
Sociodemographic				
Age, m(sd)	47.43 (12.86)	38.32 (13.01)	*t* = −2.70	0.009
Female, *n* (%)	19.00 (67.9%)	22.00 (71.0%)	ch-sq = 0.07	0.800
Male	9.00 (32.1%)	9.00 (29.0%)		
Years Education, m(sd)	15.86 (2.24)	16.00 (2.13)	*t* = 0.25	0.800
In Relationship, *n* (%)	19.00 (67.9%)	23.00 (74.2%)	ch-sq = 2.22	0.530
Ethnicity, *n* (%)			ch-sq = 3.71	0.450
White	18.00 (64.3%)	24.00 (77.4%)		
Black	3.00 (10.7%)	1.00 (3.2%)		
Hispanic	4.00 (14.3%)	4.00 (12.9%)		
Asian	3.00 (10.7%)	1.00 (3.2%)		
Other	0.00 (0.0%)	1.00 (3.2%)		
Medical				
Acute Symptoms, m (sd)	20.36 (5.79)	14.58 (4.72)	*t* = −4.22	<0.001
Current Symptoms, m (sd)	9.07 (4.98)	4.39 (3.02)	*t* = −4.31	<0.001
# Comorbidities, m (sd)	1.57 (1.37)	1.23 (1.43)	*t* = −0.95	0.350
Hospitalized, *n* (%)	4.00 (14.3%)	0.00 (0.0%)	ch-sq = 4.75	0.030
Seeking Clinical Care, *n* (%)	22.00 (78.6%)	13.00 (41.9%)	ch-sq = 8.18	0.004
Chalder Fatigue Score, m(sd)	23.89 (6.40)	19.03 (7.07)**	*t* = −2.74	0.008
Meet Criteria Chalder Fatigue, *n* (%)	24 (85.7%)	11 (35.5%)	ch-sq = 15.50	<0.001
Chalder Physical Fatigue, m(sd)	15.29 (4.63)	12.20 (4.37)**	*t* *t* = −2.61	0.010
Chalder Mental Fatigue, m(sd)	8.61 (3.20)	7.17 (2.80)**	*t* = −1.83	0.070
IADLs, m (sd)	7.25 (1.27)	7.93 (0.26)**	*t* = 2.80	<0.009
Psychiatric				
Prior Psychiatric History, *n* (%)	13 (46.4%)	14 (45.2%)	ch-sq = 0.01	0.900
PHQ-9, m(sd)	13.43 (5.15)	7.68 (5.10)	*t* = −4.31	<0.001
Meet Criteria PHQ−9, *n* (%)	22 (78.6%)	11 (35.5%)	ch-sq = 11.08	<0.001
GAD-7, m(sd)	10.54 (5.70)	4.90 (4.14)	*t* = −4.37	<0.001
Meet Criteria GAD-7, *n* (%)	15 (53.6%)	4 (12.9%)	ch-sq = 11.15	<0.001
PCL-5, m(sd)	30.50 (14.50)	15.94 (12.51)	*t* = −4.15	<0.001
Meet Criteria PCL-5, *n* (%)	13 (46.4%)	3 (9.7%)	ch-sq = 10.05	0.002
Q-LES-Q, m(sd)	45.57 (13.70)	66.90 (18.53)	*t* = 4.98	<0.001
Neuropsychological				
TOPF, m(sd)	107.06 (12.56)**	107.04 (14.22)**	*t* = −0.00	0.100
PAOF Memory, m(sd)	2.62 (0.94)	1.42 (0.81)**	*t* = −5.19	<0.001
PAOF Language, m(sd)	1.89 (1.07)	1.14 (0.86)**	*t* = −2.96	0.004
PAOF Cognitive Intellectual, m(sd)	2.28 (1.17)	1.03 (0.85)**	*t* = −4.65	<0.001
RBANS Total, m(sd)	88.04 (15.58)	98.10 (13.22)	*t* = 2.68	0.010
MoCA Total, m(sd)	24.07 (2.48)	26.74 (2.18)	*t* = 4.41	<0.001

* *p* ≤ 0.05 is significant, **Chalder Fatigue Score: Did Not Take Time Off: 19.03 (7.07) *n* = 30, **Chalder Physical Fatigue: Did Not Take Time Off: 12.20 (4.37) *n* = 30, Chalder Mental Fatigue Score: Did Not Take Time Off: 7.17 (2.80) *n* = 30, **IADL: Did Not Take Time Off: 7.93 (0.26) *n* = 29, **TOPF: Took Time Off: 107.06 (12.56) *n* = 18, **TOPF: Did Not Take Time Off: 107.04 (14.22) *n* = 23, **PAOF Memory: Did Not Take Time Off: 1.42 (0.81) *n* = 30, **PAOF Language: Did Not Take Time Off: 1.14 (0.86) *n* = 30, **PAOF Cognitive Intellectual: Did Not Take Time Off: 1.03 (0.85) *n* = 30.

#### 3.3.2. Medical Characteristics

The TTO cohort reported significantly more severe acute COVID symptoms and persistent COVID symptoms compared to the NTO cohort (Table 4 and Supplementary Figure S4). Four patients in the sample were hospitalized, all of whom were in the TTO group. Similarly, there were significantly more participants in the TTO cohort who sought clinical care for post-COVID symptoms. However, there was no significant difference in the average number of comorbidities between the two groups. Furthermore, the TTO cohort reported a significantly higher percentage of participants meeting criteria for clinically significant fatigue on the Chalder Fatigue Scale, as well as a significantly higher average Chalder Fatigue Score. Lastly, the TTO cohort reported a significantly lower average score on their ability to complete Instrumental Activities of Daily Life (IADLs) when compared to the NTO cohort.

#### 3.3.3. Psychiatric characteristics

Although there was no significant difference in psychiatric history between the two groups, there were significant differences across depression, anxiety, and PTSD screening measures (Table 4 and Supplementary Figure S4). The TTO group had on average higher scores on the self-report measure (PHQ-9), and were over twice as likely to meet criteria for clinically significant depression (78.7% vs. 35.5%) when compared to the NTO group. Similarly, the TTO group reported significantly higher levels of anxiety and PTSD symptoms, with higher average scores on the GAD-7 and PCL-5, respectively. The TTO group was 4× as likely to meet criteria for clinical anxiety (53.6% vs. 12.9%) and almost 5× as likely to meet criteria for clinically significant PTSD (46.4% vs. 9.7%) when compared to the NTO group. Moreover, the TTO cohort reported a significantly lower score on the Endicott Quality of life Scale.

#### 3.3.4. Neuropsychological characteristics

Both groups were nearly identical in terms of estimated premorbid intellectual function; however the TTO cohort reported significantly higher levels of self-reported or subjective impairment on the cognitive intellectual, memory, and language PAOF subdomains (Table 4 and Supplementary Figure S4). Consistent with their scores in subjective impairment, the TTO group had a significantly lower average total score on the RBANS, as well as the MoCA.

### 3.4. Predictors of time off

A univariate logistic regression model was developed using backward elimination to identify independent variables within each measurement domain (sociodemographic, medical, psychiatric and neuropsychological) that were the most significant independent predictors of having taken time off work beyond COVID quarantine. The most significant predictors in our model were acute COVID-19 symptom score and total MoCA score. For every 1-point increase in peak COVID symptom severity there was an 18% increased likelihood of taking time off, whereas, for each 1-point decrease in total MoCA score there was a 40% increase in the likelihood of taking time off (Table 5).

**Table 5:** Multivariate logistic regression with backward elimination predicting odds of taking time off from work after acute COVID-19 quarantine versus immediate resumption of employment after quarantine.

Variable	Odds ratio	95% Confidence interval (lower bound)	95% Confidence interval (upper bound)	*p*-value
Peak Acute COVID-19 symptom score	1.18	1.02	1.37	0.027
Total MoCA Score	0.58	0.40	0.83	0.003
Age	Removed by backwards stepwise elimination.			
Chalder Fatigue Scale Score				
Current COVID Symptom Score				
GAD-7 Score				
RBANS Total Score				

### 3.5. Comparison of performance suffered vs. performance did not suffer

#### 3.5.1. Sociodemographic characteristics

While the PS group on average was 4 years older than the PDNS group, there were no significant sociodemographic differences between the cohorts (Table 6). Additionally, a majority of participants in both groups identified as white, female and had similar levels of education. Of important note, there was minimal overlap between the PS and TTO cohorts, meaning that individuals who felt there occupational performance suffered were just as likely to have taken time off of work beyond required quarantine as not.

**Table 6:** Characteristics of participants who reported performance suffered at work due to persistent COVID symptoms compared to those who did not report their occupational performance suffered.

Variable	Performance suffered	Performance did not suffer	Statistic	*p* *
	*N* = 28	*N* = 31		
Sociodemographic				
Age, m(sd)	44.50 (12.37)	40.97 (14.66)	*t* = −0.99	0.320
Female, *n* (%)	19.00 (67.9%)	22.00 (71.0%)	ch-sq = 0.07	0.800
Male	9.00 (32.1%)	9.00 (29.0%)		
Years education, m(sd)	15.86 (2.31)	16.00 (2.07)	*t* = 0.25	0.800
In relationship, *n* (%)	18.00 (64.3%)	24.00 (77.4%)	ch-sq = 2.39	0.500
Ethnicity, *n* (%)			ch-sq = 2.45	0.650
White	20.00 (71.4%)	22.00 (71.0%)		
Black	2.00 (7.1%)	2.00 (6.5%)		
Hispanic	3.00 (10.7%)	5.00 (16.1%)		
Asian	3.00 (10.7%)	1.00 (3.2%)		
Other	0.00 (0.0%)	1.00 (3.2%)		
Medical				
Acute symptoms, m(sd)	19.50 (5.06)	15.35 (6.11)	*t* = −2.82	0.007
Current symptoms, m(sd)	8.14 (5.13)	5.23 (3.79)	*t* = −2.50	0.020
# Comorbidities, m(sd)	1.75 (1.69)	1.06 (1.00)	*t* = −1.87	0.070
Hospitalized, *n* (%)	2.00 (7.14%)	2.00 (6.5%)	ch-sq = 0.01	0.920
Seeking clinical care, *n* (%)	21.00 (75.0%)	14.00 (45.2%)	ch-sq = 5.43	0.020
Chalder fatigue score, m(sd)	23.96 (5.36)	18.97 (7.80)**	*t* = −2.86	0.006
Meet criteria chalder fatigue, *n* (%)	21 (75.0%)	14 (45.2%)	ch-sq = 5.78	0.060
Chalder physical fatigue, m(sd)	15.39 (3.70)	12.10 (5.07)**	*t* = −2.84	0.006
Chalder mental fatigue, m(sd)	8.57 (2.36)	7.20 (3.50)**	*t* = −1.76	0.080
IADLs, m(sd)	7.50 (0.88)	7.69 (1.04)**	*t* = 0.74	0.460
Psychiatric				
Prior psychiatric history, *n* (%)	14 (50.0%)	13 (41.9%)	ch-sq = 0.39	0.540
PHQ-9, m(sd)	11.68 (5.11)	9.26 (6.30)	*t* = −1.61	0.110
Meet criteria PHQ-9, *n* (%)	19 (67.9%)	14 (45.2%)	ch-sq = 3.08	0.080
GAD-7, m(sd)	8.00 (5.11)	7.19 (6.12)	*t* = −0.54	0.590
Meet criteria GAD-7, *n* (%)	9 (32.1%)	10 (32.3%)	ch-sq = 0.00	1.000
PCL-5, m(sd)	25.39 (14.02)	20.55 (16.14)	*t* = −1.23	0.230
Meet criteria PCL-5, *n* (%)	9 (32.1%)	7 (22.6%)	ch-sq = 0.68	0.410
Q-LES-Q, m(sd)	51.50 (16.06)	61.55 (21.30)	*t* = 2.03	0.050
Neuropsychological				
TOPF, m(sd)	110.62 **(10.00)	103.30 (15.53)**	*t* = −1.78	0.080
PAOF memory, m(sd)	2.21 (1.03)	1.80 (1.06)**	*t* = −1.48	0.140
PAOF language, m(sd)	1.75 (1.04)	1.27 (0.98)**	*t* = −1.82	0.070
PAOF cognitive intellectual, m(sd)	2.05 (1.02)	1.24 (1.22)**	*t* = −2.75	0.008
RBANS total, m(sd)	93.11 (14.23)	93.52 (16.15)	*t* = 0.10	0.920
MoCA total, m(sd)	25.32 (2.25)	25.61 (3.03)	*t* = 0.42	0.670

* *p* ≤ 0.05 is significant, **Chalder Fatigue Score: Performance Did Not Suffer: 18.97 (7.80) *n* = 30, **Chalder Physical Fatigue: Did Not Take Time Off: 12.10 (5.07) *n* = 30, Chalder Mental Fatigue Score: Did Not Take Time Off: 7.20 (3.50) *n* = 30,** IADLs: Performance Did Not Suffer 7.69 (1.04) *n* = 29, **TOPF: Performance Suffered: 110.62 (10.00) *n* = 21, **TOPF: Performance Did Not Suffer: 103.30 (15.53) *n* = 20, **PAOF Memory: Performance Did Not Suffer: 1.80 (1.06) *n* = 30, **PAOF Language: Performance Did Not Suffer: 1.27 (0.98) *n* = 30, **PAOF Cognitive Intellectual: Performance Did Not Suffer: 1.24 (1.22) *n* = 30.

#### 3.5.2. Medical characteristics

On average, the PS cohort reported significantly more severe acute COVID symptoms and persistent COVID symptoms when compared to the PDNS cohort (Table 6 and Supplementary Figure S5). As such, there were significantly more participants in the PS cohort who sought clinical care. Despite this, the two groups had no significant differences in the average number of medical comorbidities or the number of participants who had been hospitalized. While the PS cohort reported a significantly higher average score on the Chalder Fatigue Scale, there was no significant difference in the percentage of participants who met criteria for clinically significant fatigue. Finally, there was no significant difference between the PS and PDNS cohorts’ ability to complete instrumental activities of daily life (IADLs).

#### 3.5.3. Psychiatric characteristics

No significant difference was found between the PS and PDNS cohorts in terms of prior psychiatric history, or across screening measures for depression, anxiety, or PTSD (Table 6 and Supplementary Figure S5). In contrast, the PS cohort had lower average scores on the Endicott Quality of life Scale when compared to the PDNS cohort.

#### 3.5.4. Neuropsychological characteristics

The PS and PDNS groups did not significantly differ in estimated premorbid intellectual function, nor across neurocognitive assessments (Table 6 and Supplementary Figure S5). The only significant difference found between the groups was in subjective higher order cognitive/executive function (PAOF Cognitive Intellectual subdomain), with the PS cohort reporting significantly higher levels of subjective impairment. Subjective impairment in memory and language (PAOF Memory, PAOF Language) did not significantly differ. In contrast to self-report measures, scores on more objective neurocognitive measures (RBANS and MoCA) were nearly identical.

## 4. Discussion

These results suggest that taking time away from work beyond the required quarantine period may predict impairment in psychiatric, neurocognitive and functional status. The self-reported reasons for taking time off, most notably physical symptoms and cognitive impairment, corresponded to assessment results. The strongest independent predictors of taking time off were severity of acute COVID illness and cognitive performance as assessed by the MoCA. In contrast, subjective impairment in occupational performance among those currently working appears to focus on subjective assessment of peak and current COVID-19 symptoms, fatigue, decreased motivation, and subjective difficulty with higher cognitive function, without evidence of neuropsychological difficulty on testing. These distinctions may have important implications for assessing and treating individuals who both took extended time off of work post COVID quarantine and those who are currently working but expressing difficulty.

Individuals in the TTO cohort reported significantly greater illness severity across multiple measures, including acute and persistent COVID symptoms, hospitalizations, and clinical fatigue when compared to the NTO cohort, despite there being no significant differences in the number of medical comorbidities between the two groups. This is also reflective in the greater frequency of diminished functional capacity on their IADLs. This appears to be consistent with reports that 11.8%–50% of PASC patients experienced new or worsening impairment in activities of daily life (8, 10), while 52.3%–78% reported experiencing persistent fatigue (4, 5, 11) and that those who were hospitalized with COVID-19 have higher rates of extended time from work and disability compared to those with milder illness (5, 6, 8–10).

Despite there being no significant differences in psychiatric history between the NTO cohort and TTO cohorts, the TTO cohort was 2–5× as likely to meet clinical criteria for depression, anxiety and PTSD post-COVID and scored significantly lower on the Endicott Quality of Life scale. The increased psychiatric morbidity post-COVID may be reflective of the increased disease burden and functional impairment experienced by those who took time off, culminating in a lower quality of life. These findings are consistent with those found in a recent meta-analysis, where Zeng et al. 2022 estimated one-fifth of recovered COVID patients demonstrated psychiatric symptoms within the year after recovery, with 18.3% exhibiting symptoms of depression, 17.9% PTSD, and 16.2% anxiety. Likewise, Garrigues et al. 2020 found patients with persistent COVID symptoms post-hospitalization had altered health related quality of life outcomes across mobility, self-care, usual activities, pain/discomfort and anxiety/depression.

Concordant with the increased disease severity, functional impairment and psychiatric burden, the TTO cohort reported significantly higher degrees of subjective difficulty with cognitive function and lower objective scores across all neurocognitive assessments, despite both groups having similar levels of education and pre-morbid intellectual function. The TTO cohort not only reported significantly higher levels of subjective impairment across the memory, language and cognitive intellectual POAF subdomains, but scored significantly lower on both the RBANS and MoCA total scores. In addition, the most prevalent self-attributed reasons for taking time from work included fatigue, concentration, and memory impairment. These findings validate that the subjective impairment felt by individuals who took time off is indicative of objective neurocognitive deficits. Comparably, previous literature reports cognitive deficits and memory impairment as some of the most frequently reported and most debilitating symptoms experienced with PASC, with concentration and cognitive difficulty reported by 19.7%–55% of participants and memory impairment experienced by 17.5%–51% of participants (4, 5, 34). Our prior research indicates that extremely low neurocognitive performance is present in nearly 40% of individuals seeking care for PASC, many of whom have taken extended time off from work and report significant current neurocognitive difficulty (35).

In contrast to the analysis of those who had taken extended time off, self-reported difficulty with current employment is largely a subjective assessment without objective correlates. Those who reported occupational impairment post-COVID did report significantly more severe acute and persistent COVID symptoms, fatigue, and subjective cognitive difficulty in executive functions as well as higher rates of seeking post-COVID care when compared to those who did not report occupational impairment. However, there were no objective differences in medical, psychiatric or neurocognitive status. For instance there were no differences number of comorbidities or in the number of hospitalizations between the groups, and while there was a significantly higher average scores of fatigue, there were no significant differences in the number of individuals who met criteria for clinically significant fatigue between the two groups.

While those who felt their occupational performance suffered reported lower average scores on the subjective assessment Endicott Quality of life Scale, there were no significant differences across depression, anxiety or PTSD between those who experienced occupational impairment and those who did not. Similarly, when looking at neurocognitive characteristics, those who reported occupational impairment reported significantly higher levels of subjective cognitive impairment. However, when compared to those who did not report occupational impairment there were no significant differences in pre-morbid intellectual function, level of education, subjective memory or language impairment, nor scores on objective neurocognitive assessments as seen in the RBANS and MoCA.

When looking at reports of diminished performance among those who are currently working at the time of assessment, it appears that this complaint largely reflects subjective reports of COVID symptom burden and fatigue as well as the subjective difficulty with executive functions. This is further reflected by the most prevalent self-attributed reasons for impaired occupational performance being identified as difficulties with fatigue and with motivation. In contrast to the analysis related to taking time off, objective measures of cognitive performance were not predictive of self-reported diminished work performance. Thus, efforts to enhance subjective work performance should likely focus on enhancing motivation, and diminishing overall physical symptom burden, particularly fatigue. It is likely that these factors, often associated with cognitive complaints (4, 5, 11, 34) contribute to subjective difficulties with planning and organization even though cognitive testing may not bear this out. Motivational factors appear to be important as well, however, the significance of this finding requires clarification. Overall, it is important to emphasize that reported difficulty with work performance should not be dismissed. These individuals report diminished quality of life and should have a thorough medical, psychiatric and neurocognitive workup to address any modifiable factors. Physical symptom mitigation, attempts to treat fatigue, clarification of motivational factors and attention to specific executive function complaints should be addressed.

It is worth discussing that cognitive dysfunction, fatigue and motivational issues are not unique to PASC. Recent literature has called attention to a subset of disorders that share significant features with PASC; this includes Myalgic Encephalitis/Chronic Fatigue Syndrome (ME/CFS) and disorders of Autonomic Dysfunction (AD) such as, orthostatic intolerance and Postural Orthostatic Tachycardia Syndrome (POTS) (36–42). These disorders, while distinct, are highly correlative and consist of core symptoms of exertional intolerance, impaired functional ability, chronic fatigue and cognitive dysfunction; they are thought to stem from similar precipitating factors such as infectious illness and immune disorders (36–41). As previously discussed, cognitive complaints are often associated with diminished motivation, overall physical symptom burden and fatigue (4, 5, 11, 34). Thus it is important to note that our measure of motivation likely includes characteristics of cognitive dysfunction including apathy, executive dysfunction, and blunted emotional capacity. Given the significant overlap in symptoms between PASC and the aforementioned disorders, it is likely that the motivational issues reported with occupational impairment in the PS cohort, could be a downstream result of the fatigue and cognitive dysfunction by disease pathophysiology.

Exertional intolerance is an underlying commonality of PASC, PEM, ME/CFS, AD & POTS (36–45). Exertional intolerance is a non-specific descriptor used to encompass intolerance to any level of physical exertion or activity. This includes intolerance to exertion from minimal activity as seen in Post Exertional Malaise (PEM), as well as intolerance to exertion from the simple act of standing up seen with AD (36–46). A recent study reported that 58% (*n* = 485) of participants with PASC met criteria for ME/CFS (37), while another found that 79 out of 80 long-COVID patients met criteria for PEM (36). Similarly, another study reported AD in 61.1% long-COVID patients (47). Moreover, POTS and ME/CFS are known to be highly comorbid and have a well-documented association with cognitive impairment (41, 42).

Two potentially explanatory phenomena for the cognitive dysfunction, fatigue and motivational issues seen in PASC, are PEM and AD (37–42).While the pathophysiology of these disorders has not yet been established, circulatory impairment, chronic inflammation, auto-antibodies, neuroinflammation, elevated cytokine levels, direct viral invasion of CNS structures, and neurotransmitter dysregulation have been consistently hypothesized as potential etiologies for each the previously mention disorders (ie: AD, PEM, PASC, ME/CFS and POTS) (36–45). Many studies hypothesize that PEM and AD are potential etiologies for the neurological manifestations seen in PASC, due to the significant similarities ME/CFS and POTS share with PASC and the established correlation of chronic fatigue and cognitive impairment in these disorders, of which PEM and AD are respective hallmark features (39–42, 46–47). The significant exertional intolerance of PEM and AD, seen as a result of minimal activity, in addition to symptoms of cognitive dysfunction, and fatigue create a significant barrier to completing a typical workload and is likely to have a notable impact on work place performance.

Fatigue is the primary symptom associated with exertional intolerance (36–40). Although exertional intolerance was not directly measured in this study, the significantly increased overall fatigue, particularly, physical fatigue, indicated in both TTO and PS cohorts, suggests that physical fatigue may have been an indicator of exertional intolerance in the cohort. Despite an absence of significantly different levels of mental fatigue in the TTO and PS groups, both cohorts indicated higher levels cognitive difficulty. The TTO cohort demonstrated more severe cognitive deficits, with significant deficits in both objective and subjective measures. In contrast, the PS cohort endorsed higher levels of subjective- but not objective- cognitive impairment. This suggests that physical fatigue, not mental fatigue, may play a significant role in objective and subjective cognitive dysfunction. It could be asserted that the motivational issues and impaired occupational performance could be related to physiological issues we did not directly measure, such as AD and PEM.

There are some important limitations and strengths to the generalizability of the results in our dataset. At the time of study design, the pathophysiology of COVID-19 was still under investigation and little was known about PASC. As a result, this study did not include direct inquiry into exertional intolerance or AD. Other important limitations include a small sample size and somewhat homogenous sociodemographic characteristics of the participants. In addition, this study includes individual at higher levels of the employment spectrum and under-represents those at lower levels. We relied on retrospective reports of taking time off and the majority of participant assessments were conducted retrospectively, subsequent to participants taking time off. Particularly with respect to the TTO analyses, it cannot be determined for certain if results reflect a cause, effect or some combination. However, despite these limitations there remain some major strengths to the results of our study, which include inclusion of both community and clinic samples in our study population, and a very thorough assessment of medical, psychiatric and neurocognitive characteristics. Of particular strength is this studies use of objective neurocognitive assessments, the RBANs and MoCA, to evaluate actual cognitive performance.

In conclusion, despite its limitations, this study may shed light on factors that contribute to why individuals post-COVID take time away from work or feel their occupational performance has suffered, which is otherwise lacking from the literature to date. Furthermore, based on our clinical experience of treating many patients with PASC, we feel that our findings reflect the clinical realities. Those who took time off post-COVID beyond quarantine have persistent medical, psychiatric and neurocognitive difficulties. Such individuals appear to require close follow up to identify address modifiable factors across these domains, including attention to neurocognitive performance. In contrast, occupational impairment is most likely more reflective of subjective impairment and less substantiated by objective evidence. Such individuals still experience significantly diminished quality of life, require thorough work up for objective causes of physical symptoms, fatigue, and neurocognitive complaints. In addition, motivational factors regarding current employment should be clarified and addressed.

## Data Availability

The raw data supporting the conclusions of this article will be made available by the authors, without undue reservation.

## Supplementary material

SUPPLEMENTARY FIGURE S1

Compares the employment status, time away from work, and working hours of those who took time off work (TTO) and those who did not (NTO).

SUPPLEMENTARY FIGURE S2

Self-attributed reasons for taking time off work.

SUPPLEMENTARY FIGURE S3

Self-attributed reasons for occupational performance suffered among those currently working.

SUPPLEMENTARY FIGURE S4

Comparison of medical characteristics between TTO vs NTO and PS vs. PDNS domains. Figure 4 (**A**). Compares variables measured in mean scores. Figure 4 (**B**). Compares variables measured in percentage number. *p < 0.05 is significant.

SUPPLEMENTARY FIGURE S5

Comparison of psychiatric characteristics between TTO vs. NTO and PS vs. PDNS domains. Figure 4 (**A**). Compares average PHQ-9 scores of major depression. Figure 4 (**B**). Compares average GAD-7 scores of generalized anxiety. Figure 4 (**C**). Compares average PCL-5 scores of PTSD. Figure 4 (**D**). Compares average Q-LES-Q scores of quality of life. *p < 0.05 is significant.

SUPPLEMENTARY FIGURE S6

Comparison of neuropsychological characteristics between TTO vs. NTO and PS vs. PDNS domains. Figure 4 (**A**). Compares average subjective neurocognitive scores self-assessed cognitive function across POAF subdomains. Figure 4 (**B**). Compares average objective neurocognitive total scores on the RBANs . Figure 4 (**C**). Compares average objective neurocognitive total scores on the MoCA. *p < 0.05 is significant.

SUPPLEMENTARY DATA SHEET 2

Employment questionnaire utilized to collect information on employment status pre-COVID illness, time taken off work, length of time away from work, self-reported reasons for taking time off, current employment status, current hours working, FMLA, disability, interest in returning to work, performance at work, self-attributed reasons for impaired performance at work.
