# Supplementary material for: Impact of COVID-19 on employment: sociodemographic, medical, psychiatric and neuropsychological correlates
Source: Front Rehabil Sci. 2023 Jul 11;4:1150734. doi: 10.3389/fresc.2023.1150734 (PMC10368129; doi:10.3389/fresc.2023.1150734)
Supplement: Supplementary file 1 [file Datasheet1.zip › Supplementary Figure 6..docx]

Supplementary Material

Figure 6.

**Madison Thompson*, B.S._1_ — Stephen J Ferrando, M.D._1,2_ — Rhea Dornbush, Ph.D._1,2_ — Sean Lynch, M.D._1_,_3_ — Sivan Shahar, M.D._1,4_ — Lidia Klepacz, M.D._1,2_ — Abbas Smiley, M.D._1,5_**

*** Correspondence:** Madison Thompson: [mthompso20@student.nymc.edu](mailto:mthompso20@student.nymc.edu), Stephen Ferrando: [Stephen.Ferrando@wmchealth.org](mailto:Stephen.Ferrando@wmchealth.org)

**Supplementary Figure 6.** Comparison of neuropsychological characteristics between TTO vs. NTO and PS vs. PDNS domains. Figure 4 (A). Compares average subjective neurocognitive scores self-assessed cognitive function across POAF subdomains. Figure 4 (B). Compares average objective neurocognitive total scores on the RBANs . Figure 4 (C). Compares average objective neurocognitive total scores on the MoCA. **p* $\text{≤}$ 0.05 is significant.
