# Supplementary material for: Impact of COVID-19 on employment: sociodemographic, medical, psychiatric and neuropsychological correlates
Source: Front Rehabil Sci. 2023 Jul 11;4:1150734. doi: 10.3389/fresc.2023.1150734 (PMC10368129; doi:10.3389/fresc.2023.1150734)
Supplement: Supplementary file 1 [file Datasheet1.zip › Supplementary Figure 1. .pdf]

Supplementary Material

Figure 1.

Madison Thompson*, B.S.[1] — Stephen J Ferrando, M.D.[1,2] — Rhea Dornbush, Ph.D.[1,2] — Sean Lynch, M.D.[1,3] — Sivan Shahar, M.D.[1,4] — Lidia Klepacz, M.D.[1,2] — Abbas Smiley, M.D.[1,5]

*** Correspondence:** Madison Thompson: mthompso20@student.nymc.edu, Stephen Ferrando: Stephen.Ferrando@wmchealth.org

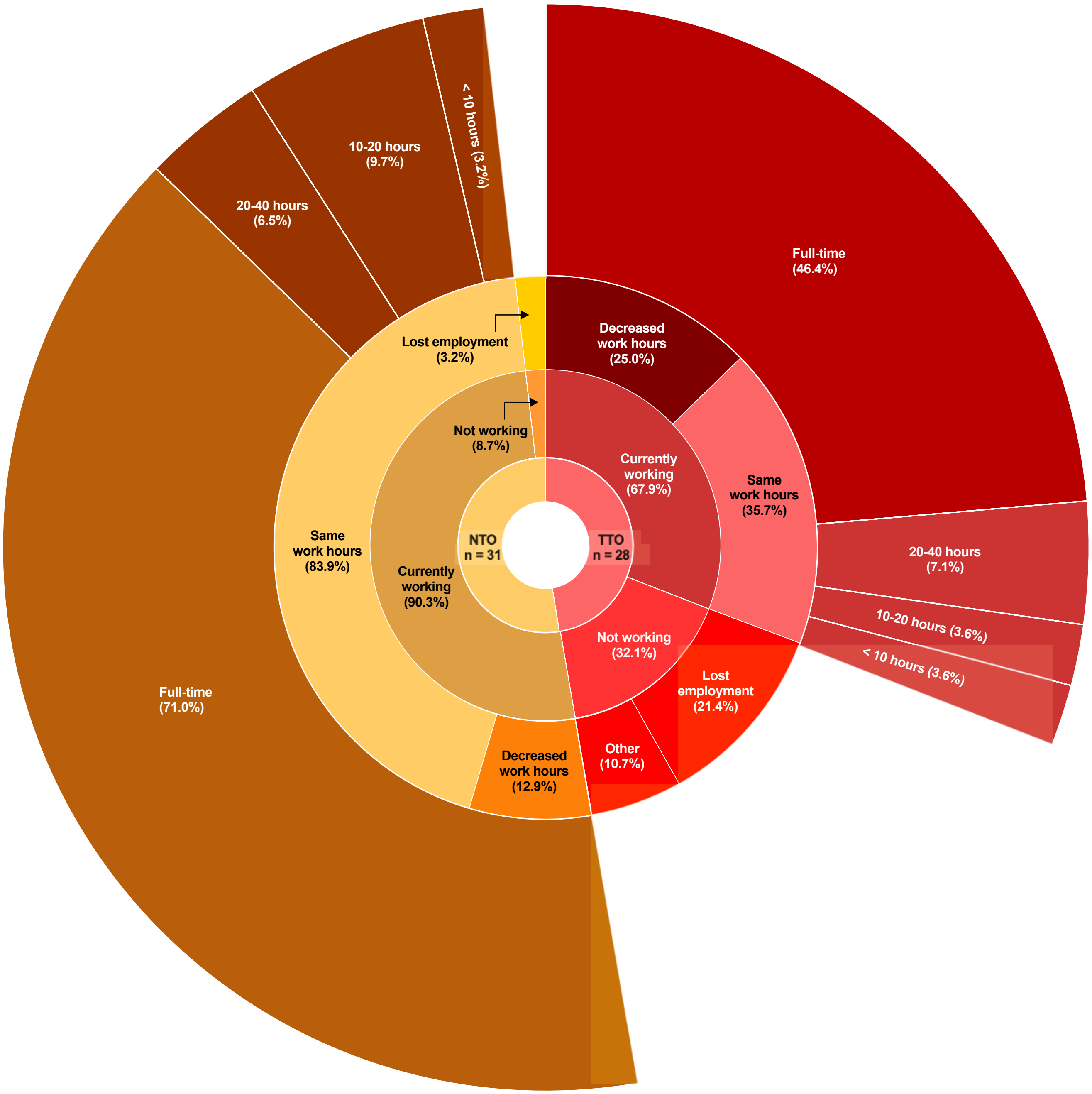

Supplementary Figure 1. Compares the employment status, time away from work, and working hours of those who took time off work (TTO) and those who did not (NTO).
