# Supplementary material for: Impact of COVID-19 on employment: sociodemographic, medical, psychiatric and neuropsychological correlates
Source: Front Rehabil Sci. 2023 Jul 11;4:1150734. doi: 10.3389/fresc.2023.1150734 (PMC10368129; doi:10.3389/fresc.2023.1150734)
Supplement: Supplementary file 2 [file Datasheet2.pdf]

Post-COVID Employment Questionnaire

WMC Health | Behavioral Health Center
Westchester Medical Center Health Network

Employment Questionnaire:

1. **Were you employed prior to your COVID-19 illness?**
    - a. Yes - Full time
    - b. Yes - Part time
    - c. No
2. **Since having COVID-19, have you needed to take time off from work due to your Post-COVID Symptoms (not due to the acute infection)?**
    - a. Yes
    - b. No
3. **If yes, how long did you have to take off?**
    - a. < 1 week
    - b. 1-4 weeks
    - c. 4-8 weeks
    - d. Greater than 8 weeks
4. **If yes, what were your reasons for taking time off?**
    - Fatigue
    - Poor concentration
    - Difficulties remembering things
    - Low motivation
    - Systemic symptoms (still feeling chills, coughing, etc.)
    - Other: ________________
5. **Did you have to take FMLA (Family/Medical Leave) time?**
    - a. Yes
    - b. No
6. **Did you file for disability?**
    - a. Yes
    - b. No
7. **If you are working, did you have to decrease the number of hours that you work?**
    - a. Yes
    - b. No

8. Are you currently working?

- a. Full time
- b. 20-40 hours
- c. 10-20 hours
- d. < 10 hours
- e. Not at all

9. If you are not working, would you like to return to work? (only answer if you are not working)

- a. Yes
- b. No

10. If yes to #9, have you taken steps to return to work?

- a. Yes
- b. No

11. If you are working, have you felt like your job performance has suffered due to Post-COVID Symptoms?

- a. Yes
- b. No

12. If yes to #10, have you noticed difficulties with any of the following while at work (circle all that apply):

- a. Concentration
- b. Attention
- c. Memory
- d. Slowness of thinking
- e. Can only do one thing at a time when I used to do multiple
- f. Fatigue/energy levels
- g. Motivation
- h. (Other) ______________________________
- i. None of the above

13. Have you been fired/laid-off/furloughed or had to quit your job due to the pandemic?

- a. Yes
- b. No

Is there anything else we should know about your experience with COVID-19 and work?

____

____

____

____
